# Synergistic Effect by Combining a gp120-Binding Protein and a gp41-Binding Antibody to Inactivate HIV-1 Virions and Inhibit HIV-1 Infection

**DOI:** 10.3390/molecules26071964

**Published:** 2021-03-31

**Authors:** Xinling Wang, Miao Cao, Yanling Wu, Wei Xu, Qian Wang, Tianlei Ying, Lu Lu, Shibo Jiang

**Affiliations:** 1 Key Laboratory of Medical Molecular Virology (MOE/NHC/CAMS), School of Basic Medical Sciences, Fudan University, Shanghai 200032, China; 18111010064@fudan.edu.cn (X.W.); 18211010033@fudan.edu.cn (M.C.); yanlingwu@fudan.edu.cn (Y.W.); xuwei0576@126.com (W.X.); wang_qian@fudan.edu.cn (Q.W.); tlying@fudan.edu.cn (T.Y.); 2 Key Laboratory of Reproduction Regulation of National Population and Family Planning Commission, The Shanghai Institute of Planned Parenthood Research, Institute of Reproduction and Development, Fudan University, Shanghai 200032, China

**Keywords:** HIV-1, gp120, gp41, antibody, combination, viral inactivation

## Abstract

Acquired immune deficiency syndrome (AIDS) has prevailed over the last 30 years. Although highly active antiretroviral therapy (HAART) has decreased mortality and efficiently controlled the progression of disease, no vaccine or curative drugs have been approved until now. A viral inactivator is expected to inactivate cell-free virions in the absence of target cells. Previously, we identified a gp120-binding protein, mD1.22, which can inactivate laboratory-adapted HIV-1. In this study, we have found that the gp41 N-terminal heptad repeat (NHR)-binding antibody D5 single-chain variable fragment (scFv) alone cannot inactivate HIV-1 at the high concentration tested. However, D5 scFv in the combination could enhance inactivation activity of mD1.22 against divergent HIV-1 strains, including HIV-1 laboratory-adapted strains, primary HIV-1 isolates, T20- and AZT-resistant strains, and LRA-reactivated virions. Combining mD1.22 and D5 scFv exhibited synergistic effect on inhibition of infection by divergent HIV-1 strains. These results suggest good potential to develop the strategy of combining a gp120-binding protein and a gp41-binding antibody for the treatment of HIV-1 infection.

## 1. Introduction

HIV-1 is a type I enveloped virus that infects CD4^+^T cells and destroys the human immune system, resulting in acquired immune deficiency syndrome (AIDS). So far, the U.S. FDA has approved more than 40 anti-HIV drugs, consisting of reverse transcriptase inhibitors (NRTIs/NNRTIs), integrase inhibitors (IIs), protease inhibitors (PIs), and entry inhibitors (EIs) [1]. However, long-term administration of antiretroviral drugs can induce drug-resistant HIV-1 strains [2,3,4]. In the viral entry process, gp120 and gp41 play different roles in receptor recognition and membrane fusion. In the receptor binding process, the interaction between gp120 and CD4 on the cell surface induces the exposure of the coreceptor binding site and enables the binding of gp120 to the coreceptor. After gp120 binding with its receptor CD4 and coreceptor CXCR4 or CCR5, gp41 conformation changes, and N-terminal heptad repeat (NHR) and C-terminal heptad repeat (CHR) interact with each other to form a hairpin-like six-helix bundle (6-HB), resulting in a core post-fusion structure that puts viral membrane and cell membrane close together for fusion, followed by viral genome release to the target cell [5,6,7]. Therefore, to inhibit the viral entry process, gp120 and gp41 could serve as potential targets for research and development of new HIV-1 entry inhibitors with improved potency.

Several entry inhibitors targeting gp120 and gp41 have been reported [8], such as protein-based inhibitors like soluble CD4 protein sCD4 [9], eCD4-IgG [10], antibodies targeting gp120 like N6 [11] and m36.4 [12], and the fusion peptide inhibitor T20 [13,14], SJ-2176 [13], T1144 [15], and C34 [7]. Soluble CD4 (sCD4), which targets the CD4-binding site (CD4bs), could inhibit HIV-1 infection [9]. mD1.22, an engineered cavity-altered single CD4 D1 domain, had a higher affinity to gp140 and higher stability than sCD4 [16]. While, 4Dm2m and 2Dm2m, each containing mD1.22, an antibody domain (m36.4) that targets CD4bs and the coreceptor-binding site (CRbs) on gp120, exhibited exceptionally potent neutralization activity against infection of divergent HIV-1 pseudoviruses [16]. eCD4-Ig, which comprises of CD4-Ig and a CCR5-mimetic sulfopeptide, showed high HIV-1 Env-binding affinity and high anti-HIV-1 potency [10]. A recombinant protein, 2DLT, consisting of CD4 D1D2 domain, a linker, and T1144, could broadly block HIV-1 entry into the target cells [17]. Besides, Griff37, a fusion protein by covalent linking of gp120-binding protein griffithsin and gp41-binding peptide C37, exhibited inhibitory activity against HIV-1 at midpicomolar level, which is much more potent than griffithsin alone [18]. These findings suggest that a viral entry inhibitor, targeting multiple sites on HIV-1 Env, is expected to have enhanced inhibition activity.

Importantly, some studies have shown that some HIV-1 entry inhibitors could inactivate cell-free virions by binding to the CD4bs in gp120 [17,19,20]. Different from the viral entry or replication inhibitors, which must act on the viruses that has attached on the cell surface or entered the target cells, viral inactivators can directly interact with viral Env protein and then inactivate virions in the absence of target cells. Therefore, a viral inactivator that can kill a virion anywhere it encounters the virion is expected to be more efficient against HIV-1 infection than a viral inhibitor that has to wait on cell surface or inside the target cell before it acts on the virion, which interacts with a receptor on the target cell or replicating in the target cell. Meanwhile, these virus inactivators can also inhibit HIV-1 infection by binding to viral Env protein, even if they could not fully inactivate the virions [21]. Our recent study has shown that a gp41-targeting peptide (e.g., T20) without HIV-1 inactivation activity could enhance the HIV-1 inactivation activity of 4Dm2m or 2Dm2m [20]. Furthermore, Ibalizumab, a CD4-directed, post-attachment inhibitor, was approved by the U.S. FDA to treat HIV-1 infection and multidrug-resistant HIV-1 infection in combination with other antiretroviral(s) [22]. These studies demonstrate that, in general, combination therapy could be an optimal way to treat AIDS.

We know that sCD4 could induce the CXCR4/CCR5 binding site and gp41 NHR exposure [19]. We also know that the NHR of gp41 is a conserved target since it is only transiently exposed after the conformational change of gp120. Therefore, since an NHR-targeting inhibitor can bind to the exposed NHR after gp120-targeting inhibitor binds to gp120, we might logically conclude that the combinatorial use of a gp120-binding protein and a gp41-binding antibody may have synergistic effect on inhibition of HIV-1 infection and inactivation of cell-free virions. We proposed to use a gp41 NHR-binding antibody D5 scFv in the combination because: (1) Antibodies have the advantage of specificity, good stability, and high affinity [23]; (2) gp41 NHR is covered by gp120 in the native conformation of HIV-1 and only transiently exposed to immune system at the fusion intermediate stage, thus being unsusceptible to mutations of resistance to the NHR-specific neutralizing antibodies. In contrast, the gp41 MPER is exposed to immune system in the native conformation of HIV-1, thus, being susceptible to mutations of resistance to the MPER-specific neutralizing antibodies, such as 10E8 [24]. The gp41 NHR-binding human antibodies D5 could neutralize HIV-1 infection by blocking the formation of fusion core [25]. D5 scFv exhibited much higher potencies than IgG molecules due to the presence of NHR-trimer steric restriction [26].

Here, we evaluated the efficacy of combining a gp120-binding protein, mD1.22, and a gp41 NHR-binding antibody, D5 scFv, on the inhibition of HIV-1 infection and, importantly, the inactivation of cell-free virions. A positive outcome from this study would imply the potential for the design and development of such antiviral drugs with bifunctional effects as inactivators of cell-free HIV-1 virions and inhibitors of HIV-1 infection.

## 2. Results

### 2.1. Combinatorial Use of mD1.22 and D5 scFv Exhibited Strong Synergism in Inhibiting Infection of HIV-1 Laboratory Strains

**Figure 1:**
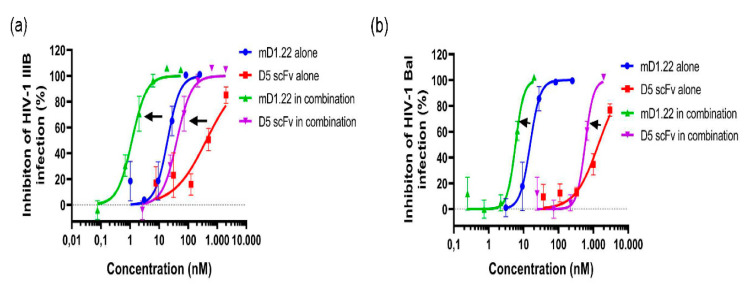
Synergism of mD1.22 combined with D5 scFv against HIV-1 laboratory-adapted strains. The blue curve and green curve represent mD1.22 used alone and in combination. The red curve and purple cure represent D5 scFv used alone and in combination. The plots represent effective concentration for inhibiting HIV-1 infection. (**a**) Synergism of mD1.22 combined with D5 scFv against laboratory-adapted HIV-1 strain IIIB (X4); (**b**) Synergism of mD1.22 combined with D5 scFv against laboratory-adapted HIV-1 strain IIIB (R5).

We first measured the inhibitory activity of mD1.22 and D5 scFv against HIV-1 laboratory-adapted strain IIIB (X4) and Bal (R5). The half-maximal inhibitory concentration (IC_50_) values of mD1.22 and D5 scFv were about 13 nM and 460 nM, respectively, when tested alone. We then mixed mD1.22 and D5 scFv at a ratio of 1:35 and again tested the inhibitory activity against HIV-1. The results showed that combining mD1.22 and D5 scFv revealed a strong synergistic effect on inhibition of infection by HIV-1 laboratory-adapted strains IIIB and Bal (Figure 1 and Table 1) with CI values of 0.16, and 0.57, respectively. Potency was enhanced 12.47-fold for mD1.22 and 11.06-fold for D5 scFv against HIV-1 IIIB infection. For HIV-1 Bal infection, the fold of enhancement of mD1.22 and D5 scFv was 2.76-fold and 2.32-fold, respectively. These results indicated that combinatorial use of the gp120-binding protein mD1.22 and the gp41-binding antibody D5 scFv could enhance anti-HIV-1 activity.

### 2.2. Combinatorial Use of mD1.22 and D5 scFv Exhibited Synergism in Inhibiting Infection by Divergent HIV-1 Strains

Meanwhile, we measured the inhibitory activity of mD1.22 and D5 scFv against primary HIV-1 isolates, T20-resistant HIV-1 strains, and AZT-resistant strains alone and in combination. Compared to the use of each alone, combinatorial usage of mD1.22 and D5 scFv showed a synergistic effect against all tested HIV-1 strains (Table 1). For primary HIV-1 isolates, the IC_50_ values of mD1.22 and D5 scFv used in combination were increased 2.42- to 22.69-fold, and 1.95- to 10.56-fold, respectively. Similar to primary HIV-1 isolates, combining mD1.22 and D5 scFv to inhibit infection by T20-resistant HIV-1 strains resulted in an enhancement of 1.55- to 2.02-fold and 1.50- to 2.29-fold, respectively. For AZT-resistant strains, mD1.22 and D5 scFv in combination exhibited strong synergism, e.g., for the strain 964, the CI value was 0.24, and enhancement of inhibitory activity was 6.99- and 7.65-fold, respectively. These results suggest that the gp41-targeting antibody has a synergistic effect with gp120-binding protein.

### 2.3. D5 scFv Enhances mD1.22-Mediated Inactivating Effect on HIV-1 Laboratory Strains and Primary HIV-1 Isolates

The previous study has shown that gp41-targeting peptides could enhance the inactivation activity mediated by gp120-targeting protein [20]. Here, we found that mD1.22 could inactivate primary HIV-1 isolates, but D5 scFv had no inactivation effect at the concentration as high as 200 nM. To explore whether the gp41-binding antibody could enhance the inactivation activity of mD1.22, we first tested the inactivation activity of mD1.22 and D5 scFv alone and in combination against cell-free virions of HIV-1 IIIB and Bal. The half-maximal effective concentration (EC_50_) values of mD1.22 alone or in combination and the fold of enhancement were showed in Table 2. mD1.22 could inactivate HIV-1 IIIB and Bal virions with the EC_50_ values of 2.09, and 9.12 nM, respectively, while in the presence of D5 scFv, the EC_50_ of mD1.22 in inactivating HIV-1 IIIB and HIV-1 Bal virions was enhanced by 2.27-fold and 4.21-fold, respectively. The 90% effective concentration (EC_90_) of mD1.22 in the combination was 1.21 nM and 4.68 nM, or 8.54-fold and 2.73-fold higher than that used alone.

Similarly, without D5 scFv, mD1.22 could efficiently inactivate HIV-1 primary isolates (91US_4 (B, R5), 97TH_NP1525 (A/E, X4, and R5) and 92UG024 (D, X4)) with EC_50_ values of 4.28-13.43 nM and EC_90_ values of 8.51-41.49 nM, respectively. As expected, such activity was enhanced by 1.15- to 5.10-fold and 0.98- to 2.43-fold when combined with D5 scFv. These results suggested that combinatorial use of the antibody binding gp41 NHR and gp120-binding protein could enhance the total inactivation effect on HIV-1 cell-free virions, both in laboratory-adapted HIV-1 strains and primary HIV-1 isolates of different subtype and tropism.

### 2.4. D5 scFv Enhances mD1.22-Mediated Inactivation Against T20-Resistant HIV-1 Strains and AZT-Resistant HIV-1 Strains

Subsequently, we investigated the inactivation activity of mD1.22 and D5 scFv alone or in combination against T20-sensitive and T20-resistant HIV-1 strains and AZT-resistant HIV-1 strains (Table 2). In the absence of D5 scFv, mD1.22 could inactivate the T20-resistant virions. The EC_50_ values were 2.56 nM and 5.08 nM for (D36G) N42T/ N43K and (D36G) V38A/N42T strains, and the EC_90_ values were 3.82 nM, and 10.03 nM, respectively. However, in the presence of D5 scFv, activity levels increased by 3.41-fold and 1.04-fold.

To determine if the mD1.22 and gp41-targeting antibody could inactivate cell-free particles of the AZT-resistant HIV-1 strains, we detected the activity alone or in combination. As shown in Table 2, mD1.22 had an inactivation effect, while D5 scFv had none. On the other hand, the combinatorial use of mD1.22 and D5 scFv could enhance the inactivation of mD1.22. The EC_50_ and EC_90_ in inactivating HIV-1 964 strain were enhanced by 1.04- and 6.37-fold, respectively. The above results indicate that the gp41-targeting antibody could significantly increase mD1.22-mediated inactivation activity against both T20- and AZT-resistant strains.

### 2.5. D5 scFv Enhances the Inactivation Activity of mD1.22 against the LRA-Reactivated HIV-1 Virions

**Figure 2:**
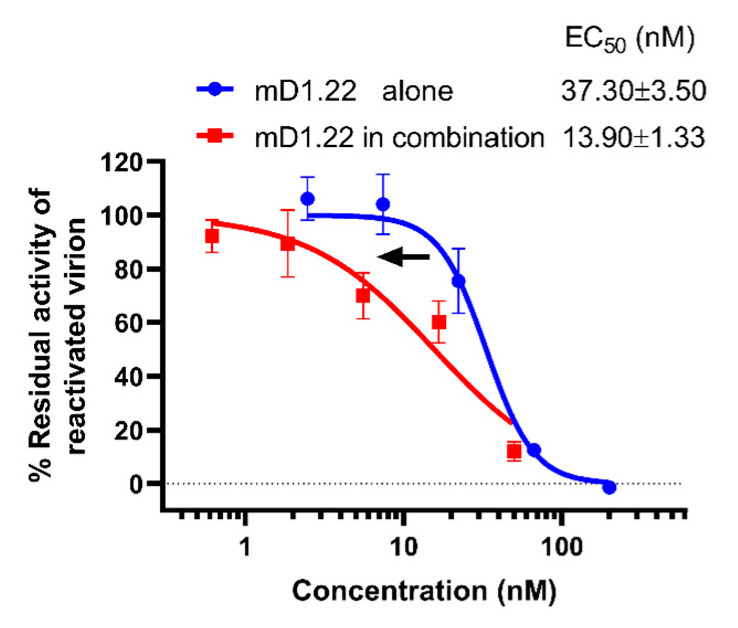
The inactivation activity of mD1.22 used alone and in combination with D5 scFv against the LRA-reactivated HIV-1 virions. Blue and red curves represent mD1.22 used alone and in combination, respectively. The samples were tested in triplicate and data were presented in mean ± SD.

We, next, tested the inactivation activity of mD1.22 against a latency-reversing agent (LRA)-reactivated HIV-1 virions. First, we used an LRA romidepsin to reactive the latent HV-1 virus in ACH-2 cells and collected the released LRA-reactivated HIV-1 particles [27]. In the absence of D5 scFv, mD1.22 could inactivate the virions released from ACH-2 cells with EC_50_ of 37.3 nM (Figure 2). However, in combination with D5 scFv, mD1.22 was more efficient with EC_50_ of 13.9 nM, which was 1.68-fold over that of mD1.22 used alone. This suggests that the gp120-binding protein mD1.22 can inactivate the reactivated latent HIV-1 virions with heightened potency in combination and that the antibody targeting gp41 NHR region exhibited a synergistic inactivation effect with mD1.22.

## 3. Discussion

Over 30 years have passed since HIV-1 was identified as the pathogen of AIDS [28]. Until now, a total of 75.7 million individuals have been infected with HIV, and about 38 million people are still living with HIV (https://www.unaids.org/en/resources/fact-sheet; accessed on 1 March 2021). Due to the promotion and application of HAART, the morbidity and mortality of HIV-infected patients were decreased drastically [29,30]. However, the efficiency of HAART was remarkably compromised by poor patient compliance, adverse effect, and drug resistance [31,32]. Moreover, NRTIs/NNRTIs, PIs, and IIs only take effect after virus entry into target cells and have no effect on cell-free virions in the blood. A previous study reported that sCD4 was able to inactivate HIV-1 virions and inhibit infection by primary HIV-1 isolates [9]. Nonetheless, a low concentration of sCD4 could, in fact, enhance HIV-1 infection in CD4^−^CCR5^+^ cells, and the affinity with gp120 limits its application [19]. Still, based on sCD4, CD4-IgG2 (PRO 542), comprised of the D1D2 domain of CD4, showed high affinity [33], and CD4-IgG2 could neutralize more primary isolates than sCD4 alone [34], and it could protect the hu-PBL-SCID mouse from infection of primary HIV-1 isolates [35]. Advanced patients treated with 25 mg/mL of CD4-IgG2 could result in 80% response with a reduction in viral load of approximately 0.5 log_10_ after treatment for 4–6 weeks [36]. The clinical trial data of CD4-IgG2 revealed the possibility of continued development, even though the potential to enhance infection of some HIV-1 strains did limit its application. The mD1.22, based on the D1 domain, had a higher affinity with gp120 and neutralizing activity than D1D2 [16], and it possesses inactivation activity against HIV-1 laboratory-adapted strains [20].

In this study, we identified the synergistic effect of combining gp120-binding protein mD1.22 and gp41-binding antibody D5 scFv on inhibition of infection by divergent HIV-1 strains, including laboratory-adapted strains, primary HIV-1 isolates, T20-resistant HIV-1 strains, and AZT-resistant HIV-1 strains. Meanwhile, we found that mD1.22 could effectively inactivate the virions of several laboratory-adapted strains, primary HIV-1 isolates, T20- and AZT-resistant HIV-1 strains, at low nanomolar level. However, the gp41-binding antibody D5 scFv could not inactivate these HIV-1 virions tested. Similar results were found in LRA-reactivated HIV-1 virions released from latent infected ACH-2 cells. Surprisingly, however, the use of D5 scFv in combination with mD1.22 could enhance the inactivation effect of mD1.22 on all the above HIV-1 strains tested in a manner similar to the previous finding in which gp41-targeting peptides (e.g., T20) could enhance 4Dm2m and 2Dm2m-mediated inactivation [20]. However, different from 4Dm2m and 2Dm2m which can bind both CD4-binding site (CD4bs) and coreceptor binding site (CoRbs) in gp120, mD1.22 could bind only to CD4bs, but not CoRbs. Therefore, the mechanism by which D5 scFv, a gp41-targeting antibody, enhances the HIV-1 inactivation activity mediated by the gp120-targeting protein mD1.22 is different from that by which a gp41-targeting peptide enhances the HIV-1 inactivation activity mediated by the gp120-targeting proteins 4Dm2m and 2Dm2m.

**Figure 3:**
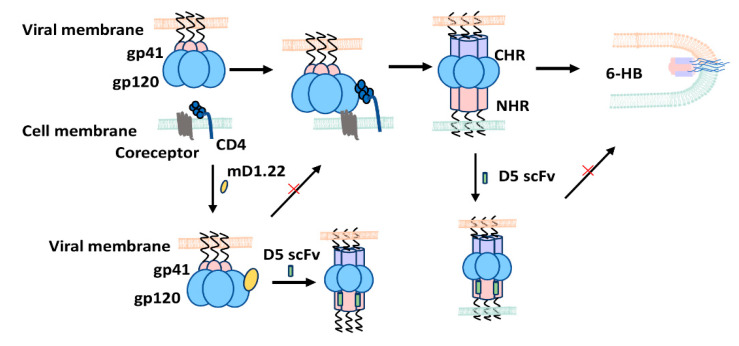
Putative mechanism of mD1.22 and D5 scFv in combination to inactivate HIV-1 virions and inhibit HIV-1 infection of the target cell. (Left) mD1.22 binds to the CD4-binding site on gp120, resulting in the partial inactivation of HIV-1 virions. Following this, binding induces several conformational changes to expose gp41 NHR trimer, which can be bound by D5 scFv, resulting in the increased inactivation of HIV-1 virions. (Right) D5 scFv can bind to exposed gp41 NHR trimer induced by CD4 on the cell surface during the viral entry process.

The conformational changes of gp120 and gp41 induced by the recognition and binding between gp120 and CD4 receptor and coreceptor CCR5/CXCR4 represents the essential process for HIV-1 entry into the target cell. The sCD4 binding to gp120 could induce conformational changes to expose the coreceptor binding site and induce the formation of the gp41 pre-hairpin fusion intermediate (PFI) with NHR grooves exposed [19]. Similar to sCD4, mD1.22 can also bind to gp120 and then induce CoRbs and the gp41 NHR groove. In parallel, D5 scFv can target the hydrophobic pocket of the gp41 NHR region and bind to the mD1.22-induced gp41 NHR trimer. This binding can stabilize mD1.22-triggered premature conformation, induce an irreversible conformational change of HIV-1 Env, and lead to disablement of the virus. Therefore, it follows that combining mD1.22 with D5 scFv exhibits a synergism, whereby, D5 scFv is able to enhance the inactivation potency of mD1.22 on HIV-1 strains (Figure 3).

As expected, besides synergistic inactivation of HIV-1 virions, the combinatorial strategy also exhibited a synergistic effect on inhibitory activity. The IC_50_ values of mD1.22 and D5 scFv in combination against different HIV-1 strains were significantly enhanced, increasing from 1.55- to 22.69-fold, and 1.37- to 11.06-fold, respectively. Except for D5 scFv that could bind with gp41, NHR trimer triggered by mD1.22, D5 scFv can also independently bind to the NHR of HIV-1 gp41 induced by cell-surface CD4 receptor during viral infection. Consequently, the process of 6-HB formation can be interrupted, thereby impeding viral-cell membrane fusion and causing inhibition of HIV-1 fusion with target cells (Figure 3). Thus, taking the unique mechanism of gp120-binding protein and gp41-binding antibody in combination, we see that the resultant synergism promotes more effective inhibitory activity against different HIV-1 strains than either the gp120-binding protein or the gp41-binding antibody used alone. Moreover, the dual synergistic effect in inactivating cell-free HIV-1 virions and inhibiting HIV-1 infection of target cells make this combinational strategy a promising candidate for further therapeutic development.

## 4. Materials and Methods

### 4.1. Cells, Virus and Proteins

MT-2 cells, ACH-2 cells, CEMx174 5.25 M7 cells, and TZM-bl cells were cultured with RPMI-1640 medium containing 10% Fetal bovine serum. Expi293 cells were cultured with SMM293-TII (Sino Biological, China). All HIV-1 strains used in this study were obtained from the National Institutes of Health AIDS Reagent Program, including HIV-1 laboratory-adapted strains, T20-resistant strains, primary HIV-1 isolates, and AZT-resistant HIV-1 strains. Fusion protein mD1.22 and D5 scFv were expressed by Expi293 cells. Briefly, the plasmids encoding mD1.22 and D5 scFv were transfected into Expi293 cells using the EZtrans transfection reagent. Then SMS 293-SUPI cell culture supplement (Sino Biological, Beijing, China) was added at a final concentration of 1% and cultured for an additional 4 days. The supernatants containing protein were collected and centrifuged at 3.000 g for 10 min. Finally, the protein was purified with Ni-NTA smart beads.

### 4.2. Inactivation of HIV-1 Virions

The inactivation activity of a test protein or antibody against HIV-1 was detected, as previously described [17,20] with minor modification. For test of mD1.22 or D5 scFv alone, the serially diluted mD1.22 (starting at 200 nM) or D5 scFv (starting at 200 nM) was mixed with an equal volume of HIV-1 (500 TCID_50_) and incubated at 4 °C for 1 h. For testing the mD1.22/D5 scFv combination, mD1.22 at 50 nM and D5 scFv at 50 nM were mixed together, followed by a series of dilution of the mixture with RPMI 1640 medium, and then incubated with an equal volume of HIV-1 (500 TCID_50_) at 4 °C for 1h. PEG6000 was then added to the culture at the final concentration of 3% and incubated at 4 °C for an additional 1 h. Afterwards, the protein-virus mixtures were centrifuged at 13.000 rpm at 4 °C for 30 min, and the virions were precipitated at the bottom of the tubes. Then the virions were washed with washing buffer (10 mg/mL BSA + 3% PEG6000) three times to remove proteins. After washing, the pellets were suspended with 100 μL RPMI 1640 medium containing 10% FBS. A total of 1 × 10^4^ MT-2 cells (used for X4 virus infection), CEMx 174 5.25 M7 cells, or TZM-bl cells (used for R5 virus) were seeded into a 96-well cell culture plate and cultured at 37 °C. For TZM-bl cells, the supernatants were removed after three days, washed with PBS, and lysed with lysis buffer (Promega, Madison, WI, USA). Then the luciferase value was measured by the luciferase assay system (Promega, Madison, WI, USA). For MT-2 cells or CEMx174 5.25 M7 cells, the culture medium containing the virus was collected at four days post-infection and mixed with an equal volume of 5% Triton-X-100. Then, the p24 antigen level was detected by ELISA assay.

### 4.3. Inhibition of HIV-1 Infection

The activity of mD1.22, D5 scFv, and their combinatorial use in inhibiting HIV-1 infection was determined as previously [37]. For testing each of them alone, mD1.22 or D5 scFv was serially diluted with RPMI 1640 medium without FBS and mixed with 100 TCID_50_ of virus. The protein-virus mixture was incubated at 37 °C for 30 min, and then 1 × 10^4^ corresponding cells were added. MT-2 cells and CEMx174 5.25 M7 cells were used as target cells for the X4, and R5 virus, respectively. For testing mD1.22/D5 scFv combination, the ratio of mD1.22 and D5 scFv in the combination should be first pre-determined based on their IC_50_ values when each of them was tested alone, following a principle of “equal potency”. For example, the IC_50_s of mD1.22 and D5 scFv against HIV-1 IIIB infection were 13, and 460 nM, respectively, mD1.22 and D5 scFv would be mixed at a ratio of 1:35 for detection of their combination inhibitory effect against HIV-1 IIIB infection. Then, the mixture was serially diluted and incubated with 100 TCID_50_ of virus and tested in a manner similar to that of protein alone. Inhibitory activity was determined by detecting the p24 antigen level in the supernatant.

ELISA assay was conducted according to the previous study [5]. Briefly, human HIV-1 IgG was coated on an ELISA plate at 5 μg/mL at 4 °C overnight and washed with PBST. Then the HIV-1 IgG was blocked with 2% nonfat milk at 37 °C for 2 h. The collected supernatants were added to the plate and incubated at 37 °C for 1 h. After washing with PBST three times, mouse anti-p24 monoclonal antibody 183 was added and incubated for another 1 h. Following this, HRP-conjugated Rabbit anti-mouse IgG was used to detect p24 antigen levels. The IC_50_ values and combination index (CI) were computed using Chou-Talalay software (Biosoft, Ferguson, MO, USA).

### 4.4. Detecting the Ability of mD1.22 to Inactivate LRA-Reactivated HIV-1 Virions Released from ACH-2 Cells

The reactivation and inactivation assays were performed according to the previous study [20]. First, HIV-1 latent infected ACH-2 cells were treated with Romidepsin [27] at a final concentration of 20 nM for 24 h. Then the supernatant containing reactivated virus was collected and centrifugated at 3.000 rpm for 10 min. The reactivated HIV-1 virus was diluted with RPMI 1640 medium without FBS. For testing each of them alone, the serially diluted mD1.22 or D5 scFv was mixed with the reactivated virus and incubated at 4 °C. For the combinatorial inactivation assay, the reactivated virus was added to the mixtures of mD1.22 (starting: 50 nM) and D5 scFv (starting: 50 nM) and incubated at 4 °C. The subsequent procedures were the same as those in Section 4.2.

### 4.5. Combination Index (CI)

The combination index of mD1.22 and D5 scFv against different HIV-1 strains was calculated using the CalcuSyn program (Biosoft, Ferguson, MO, USA). The synergistic effect was assessed according to the previous report [20]. For CI, <1, =1, and >1 represent the effect of synergism, additive effect, and antagonism, respectively. CI < 0.1 indicates very strong synergistic effect, 0.1 < CI < 0.3 means strong synergism, 0.3 < CI < 0.7 means synergism, 0.7 < CI < 0.85 means moderate synergism, and CI from 0.85 to 0.9 means slight synergism. Dose reduction and fold of enhancement were calculated as IC_50_ values tested alone/IC_50_ values tested in combination, according to the previous study [38].

## 5. Conclusions

In conclusion, this study has demonstrated that gp120-binding protein, mD1.22, can inactivate cell-free HIV-1 virions of laboratory-adapted and primary HIV-1 strains, the T20- and AZT-resistant HIV-1 strains, and LRA-reactivated virus released from the HIV-1-latently infected cells. Importantly, gp41-targeting antibody, D5 scFv, can enhance the inactivation activity of mD1.22, while D5 scFv alone has no inactivation ability against HIV-1 cell-free virions. These results demonstrated that gp41-targeting antibody D5 scFv in combination with gp120-binding protein mD1.22 exhibited a synergistic effect on inhibition of HIV-1 infection, while D5 scFv could enhance mD1.22-mediated HIV-1 inactivation effect, suggesting a good potential to develop this combinatorial strategy for treatment of HIV-1 infection.

## Figures and Tables

**Table 1:** Combinatorial use of mD1.22 and D5 scFv to inhibit infection by HIV-1 laboratory-adapted strains, primary HIV-1 isolates, as well as T20-resistant and AZT-resistant HIV-1 strains.

Virus Strain	CI	mD1.22			D5 scFv		
		IC_50_ (nM)		Fold of Enhancement	IC_50_ (nM)		Fold of Enhancement
		Alone	In Combination		Alone	In Combination	
HIV-1 Laboratory Strains							
IIIB (X4)	0.16	16.57	1.23	12.47	518.03	42.97	11.06
Bal (R5)	0.57	13.49	3.59	2.76	1190.51	359.11	2.32
HIV-1 Primary Isolates							
91US_4	0.59	26.88	7.87	2.42	674.25	196.81	2.43
92UG024	0.38	7.58	0.32	22.69	318.82	108.01	1.95
NP1525	0.20	22.97	2.66	7.64	3070.19	265.68	10.56
T20-Resistant HIV-1 Strains							
HIV-1 NL4-3 D36G (WT)	0.47	14.00	2.71	4.17	386.82	108.42	2.57
(D36G) N42T, N43K	0.79	14.58	5.72	1.55	286.19	114.47	1.50
(D36G) V38A, N42T	0.64	12.69	4.20	2.02	345.41	105.01	2.29
AZT-Resistant HIV-1 Strains							
629	0.79	17.34	6.44	1.69	764.82	322.18	1.37
964	0.24	7.19	0.90	6.99	389.64	45.03	7.65

Note: Presented data are means of three triplicates. The combination index (CI) value <1 means synergistic effect.

**Table 2:** Enhancement of inactivation activity of mD1.22 by D5 scFv against HIV-1 laboratory-adapted strains, primary HIV-1 isolates, T20-resistant- and AZT-resistant HIV-1 strains.

Virus Strains		Concentration (nM) of mD1.22		Fold of Enhancement
		Alone	In Combination	
HIV-1 Laboratory-Adapted Strains				
IIIB (X4)	EC_50_	2.09	0.64	2.27
	EC_90_	11.54	1.21	8.54
Bal (R5)	EC_50_	9.12	1.75	4.21
	EC_90_	17.46	4.68	2.73
Primary HIV-1 Isolates				
91US_4	EC_50_	4.28	1.99	1.15
	EC_90_	8.51	3.80	1.24
97TH_NP1525	EC_50_	5.92	2.51	1.36
	EC_90_	31.23	15.74	0.98
92UG024	EC_50_	13.43	2.20	5.10
	EC_90_	41.49	12.09	2.43
T20-Resistant HIV-1 Strains				
HIV-1 NL4-1 D36G (WT)	EC_50_	1.10	0.66	0.67
	EC_90_	2.12	1.16	0.83
(D36G) N42T, N43K	EC_50_	2.56	0.58	3.41
	EC_90_	3.82	1.24	2.08
(D36G) V38A, N42T	EC_50_	5.08	2.49	1.04
	EC_90_	10.03	6.28	0.60
AZT- Resistant HIV-1 Strains				
629	EC_50_	11.28	4.78	1.36
	EC_90_	20.08	8.84	1.27
964	EC_50_	3.19	1.56	1.04
	EC_90_	23.80	3.23	6.37

Note: Presented data are means of three triplicates.

## Data Availability

Not applicable.

## Sample Availability

Samples of the protein mD1.22 and antibody D5 scFv are available from the authors.
